# Comparison of three Methods of applying high flow nasal oxygen: *in vitro *study

**DOI:** 10.1186/cc14345

**Published:** 2015-03-16

**Authors:** M Muñoz Garach, O Olga, ME Yuste Osorio, R Fernandez Fernandez, R Ramirez Puerta, F Acosta Díaz, AM Perez Bailón, S Narbona Galdó, L Peñas Maldonado

**Affiliations:** 1 Hospital Universitario San Cecilio, Granada, Spain; 2 Hospital Nuestra Señora del Prado, Talavera de la Reina, Spain

## Introduction

High flow nasal (HNF) requires precise control of the fraction of inspired oxygen (FiO_2_) and flow contributed as well as an adequate adjustment of temperature and humidity of the gas provided. There are several equipments for HNF. We evaluated the FiO_2_ and flow supplied with three different systems

## Methods

There have been analyzed: (1) 'Oxygen Therapy' from Dräger Evita-XL®; (2) Fisher & Paykel Airvo® option; and (3) pack of flowmeters Debson®. Measurements were made in the distal part of the circuit that is used in clinical practice. Variables: programmed and measured FiO_2_, programmed and measured flow. We used the Oxygen Monitor Ohmeda 5120® and Flow Meter® Fisher-Porter. Before each measurement we checked and/or calibrated each of them. All measurements were performed at room temperature in the ICU of our hospital (23 to 26º).The data were processed using SPSS v.15.0.1, accepting a significance level of 95%.

## Results

**Figure 1:**
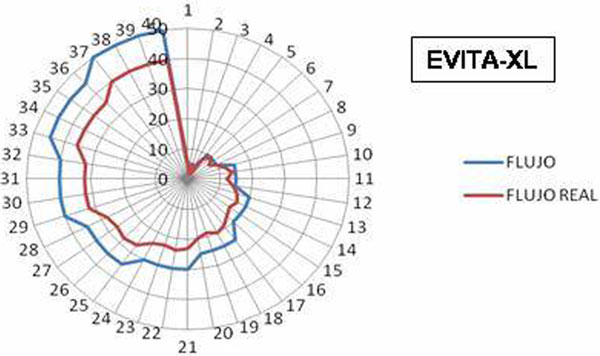
**Programmed and measured flow**.

(1) FiO_2_ variation -0.001 ± 0.09 (-0.01 to 0.002); FiO_2_ percentage variation -0.012 ± 1.88 (-0.27 to 0.25); *r*^2 ^= 0.999 and *r *= 0.998 (*P *< 0.000). Flow variation (l/minute) 5.45 ± 3.23 (4.94 to 5.96); flow percentage variation 19.59 ± 11.63 (17.75 to 21.43); *r *= 0. 997 and *r*^2^ = 0.994 (*P *< 0.000). (2) FiO_2_ variation -0.007 ± 0.26 (-0.011/-0.003); FiO_2 _percentage variation -1.4040 ± 4.73 (-2.15 to -0.67); *r *= 0.996 and *r*^2^ = 0.992 (*P *< 0.000). Flow variation (l/minute) 3.82 ± 3.85 (3.04 to 4.69); flow percentage variation 9.76 ± 8.08 (8.11 to 11.41); *r *= 0.969 and *r*^2 ^= 0.939 (*P *< 0.000). (3) FiO_2_ variation -0.005 ± 0.26 (-0001 to 0009); FiO_2_ percentage variation -0.72 ± 5.2 (-1.5 to 0.1); *r *= 0.996 and *r*^2^ = 0.992 (*P *< 0.000). Flow variation (l/minute) 3.91 ± 1.26 (3.69 to 4.13); flow percentage variation 12.77 ± 5.33 (11.84 to 13.7); *r *= 0.996 and *r*^2^ = 0.992 (*P *< 0.000). See Figure 1.

## Conclusion

The FiO_2_ percentage variation in the Airvo® is higher than the other two devices, with no clinical relevance. The flow percentage variation of Evita XL® is superior to the other two devices; this may have some clinical relevance.

